# Benefits of early application of pelvic circumferential compression device to reduce bleeding in pelvic fractures

**DOI:** 10.1186/s12891-022-05166-3

**Published:** 2022-03-03

**Authors:** Visit Rungsinaporn, Pawin Akkarawanit, Pinkawas Kongmalai

**Affiliations:** Department of Orthopaedics, Faculty of Medicine, Srinakharinwirot University, 62 Moo 7, Rangsit-Nakhon Nayok Road, Ongkharak, Nakhon Nayok, 26120 Thailand

**Keywords:** Pelvic fracture, Pelvic binder, PCCD, Hematocrit change, Blood transfusion

## Abstract

**Background:**

To study of efficacy of early pelvic circumferential compression device using in patients with suspected pelvic trauma, compared with conventional stepwise approach.

**Methods:**

Traumatic injury and at least one of the following criteria are required for inclusion: loss of consciousness or a Glasgow coma score (GCS) of less than 13; systolic blood pressure less than 90 mmHg; falling from more than 6 m; injury to several important organs; and a positive pelvic compression test. Patients who satisfied the inclusion criteria for the experimental group were given an early application of a commercial pelvic sling beginning in July 2019. The control group consisted of cases who got the device after clinical or radiological confirmation of a pelvic fracture in the previous year. Gender, age, mechanism of injury, GCS, hospital stay, amount of packed red blood cell transfusion, hematocrit in emergency room, and hematocrit 24 h after application of pelvic binder were all assessed and compared.

**Results:**

The study had a total of 30 participants, with 15 in each group. The number of packed red blood cell transfusions in the early pelvic binder group (0.80 ± 1.42) is considerably lower than in the control group (2.4 ± 2.32) (*P* = 0.008), although the hematocrit change is not statistically different between the groups (2.1 VS 0.7) (*P* = 0.191). The time it took to install a pelvic binder was considerably shorter in the early pelvic binder group (16.40 ± 5.45) than in the control group (40.40 ± 13.64) (*P* = 0.001). There were no problems associated to soft tissue and skin necrosis in either group of patients.

**Conclusions:**

The use of the PCCD for 24 h prior to clinical and radiographic confirmation has significantly reduced the rate of packed red blood cell transfusion in any pelvic fracture patient without device-related complications.

**Trial registration:**

The study was entered into the Thai Clinical Trials Registry (TCTR20210809007).

## Background

The estimated annual incidence of pelvic injury is about 23 per 100,000 people, with a substantial mortality and morbidity burden [1, 2]. Because the pelvis is so close to important blood vessels and organs, shearing of the vasculature can induce life-threatening retroperitoneal hemorrhage, as well as bleeding from broken bone ends, which necessitates immediate care [3].

The use of a pelvic circumferential compression device (PCCD) has become standard emergency therapy for trauma patients with suspected pelvic fractures, both in the pre-hospital setting [4] and in the emergency room [5]. Because of the decrease in pelvic volume, reducing and stabilizing the pelvic ring can limit fracture bleeding, resulting in favorable physiological consequences and ultimately desirable patient outcomes. However, the merits and risks of early implementation of the PCCD are still being debated [6, 7].

The current study sought to determine the efficacy of early PCCD use in patients at high risk of pelvic fracture. The goal of this study is to compare the effect of early PCCD deployment on hemorrhage control to the traditional technique. We hypothesized that using the PCCD early lowers hemorrhage and thus the requirement for transfusions in individuals with high-risk pelvic fractures.

## Materials and methods

**Fig. 1:**
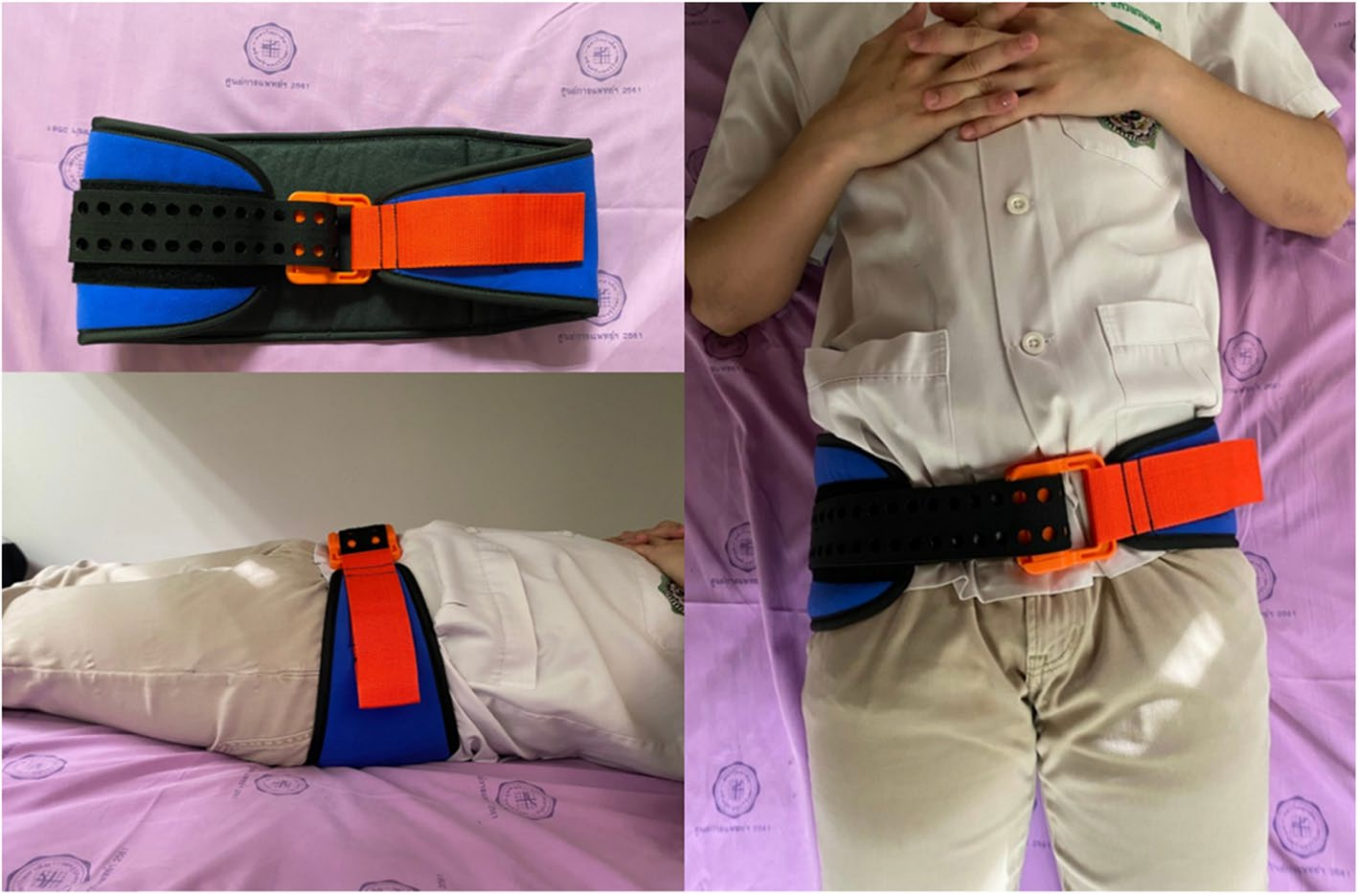
The SAM Sling® (SAM Medical Products, Wilsonville, OR, USA)

The study was entered into the Thai Clinical Trials Registry on 09/08/2021 (TCTR20210809007). The Strategic Wisdom and Research Institute Srinakharinwirot University review board (SWUEC-044/2563F) accepted the study protocol, and the manuscript was produced in compliance with the standards for Strengthening the Reporting of Observational Studies in Epidemiology (STROBE) [8]. All methods were performed in accordance with the relevant guidelines and regulations. Each patient provided written informed consent. Patients who met the criteria were enrolled and received early pelvic binder use for the emergency management of suspected pelvic trauma as they arrived at H.R.H. Maha Chakri Sirindhorn Medical Center (MSMC), Srinakharinwirot University, from July 2019 to July 2021. Traumatic injury and at least one of the following criteria were required for inclusion: loss of consciousness or a Glasgow coma score (GCS) of < 13; systolic blood pressure of < 90 mmHg; falling from > 6 m; injury to multiple vital organs; and positive pelvic compression test. If a patient met the inclusion criteria, the SAM Sling® (SAM Medical Products, Wilsonville, OR, USA), a commercially proven circumferential pelvic belt [9, 10], was worn immediately and removed 24 h later or until a definitive pelvic fracture fixation by an orthopedic surgeon. (Fig. 1).

**Fig. 2:**
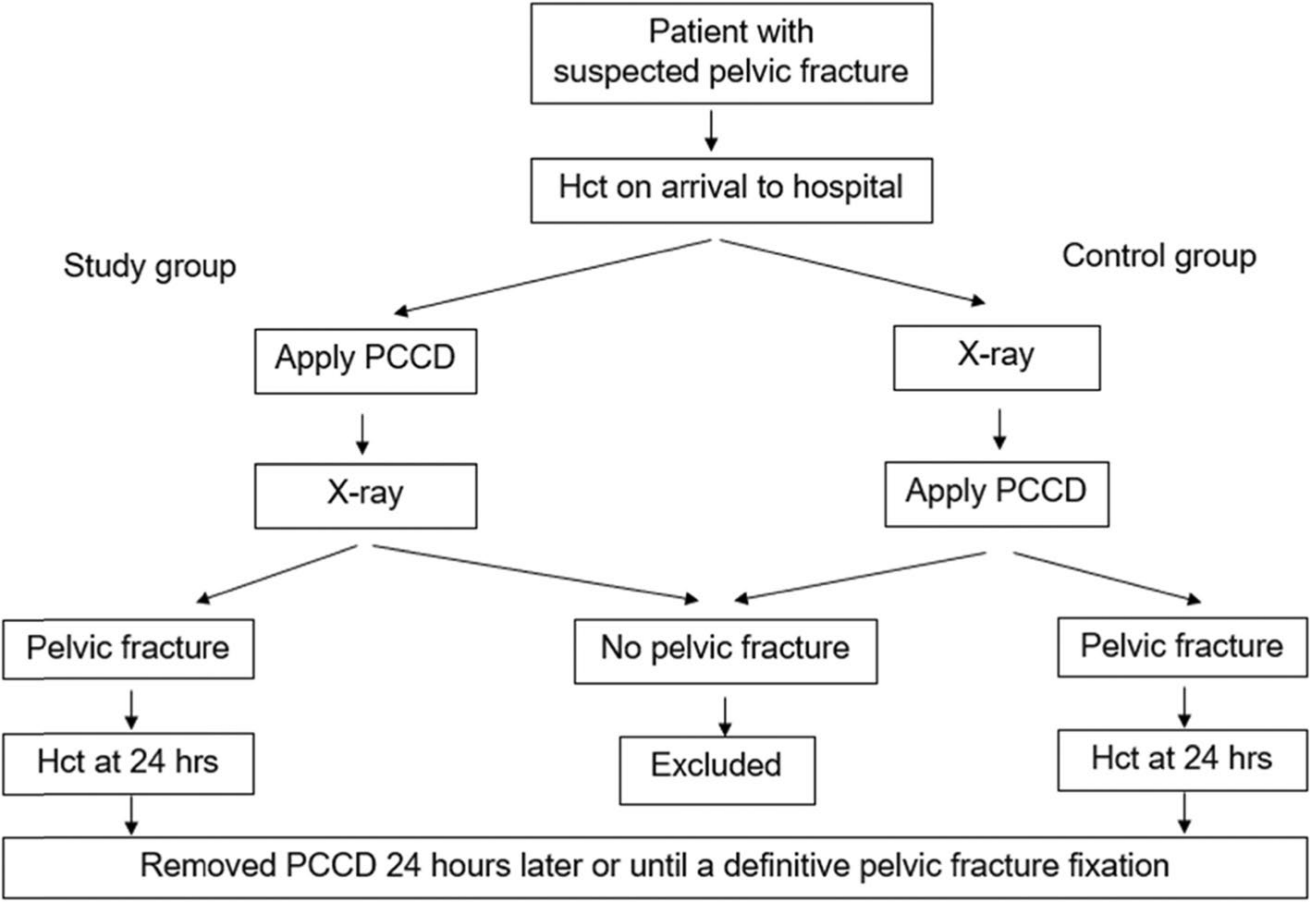
Study protocol

We compared the features of the study group patients to those of the historical control group patients, for whom pelvic binders were solely used as a standard stepwise strategy after clinical or radiographic confirmation of a pelvic fracture between July 2016 and November 2018. (Fig. 2) Age, gender, body mass index (BMI), duration during X-ray, duration to receive pelvic sling, Young and Burgess classification, surgery type, hospital stay, amount of packed red blood cell transfusion, complication after use pelvic sling, and hematocrit (Hct) at emergency room, 24-h after received pelvic sling were obtained for all participants.

## Statistical analysis

Basic descriptive statistics for categorical data were presented as frequency and percentage, while continuous data were reported as mean and standard deviation in the normal distribution and median and interquartile range in the abnormal distribution. The hematocrit, hospital stays, time to receive X-ray, time to apply binder, and amount of packed red blood cell transfusion were all analyzed using the Mann-Whitney test and the independent t-test. SPSS statistics version 22 was used to analyze the data, and statistical significance was defined as a *p*-value less than 0.05.

## Results

A total of 30 patients were enrolled in the trial, with 15 in each group. Patients in both groups were similar in terms of age, gender, weight, height, BMI, type of fracture, and definitive treatment. (Table 1) The majority of the patients were classed as having lateral compression type I. (57%). Eight patients in the early pelvic sling group had surgery, whereas four individuals in the standard pelvic sling group did. Demographic data were similar in two groups (Table 1).

**Table 1:** Demographic Data

Parameters	Early pelvic sling case (*n* = 15)	Standard pelvic sling case (*n* = 15)	*P*-value
Age (years)			0.484^a^
- Mean ± SD	49.87 ± 22.07	43.67 ± 25.70	
- Median (IQR)	46 (30, 73)	39 (18, 67)	
Male/Female	9/6	4/11	0.139^c^
Body weight (kg)			
- Mean ± SD	64.65 ± 7.32	61.73 ± 7.78	0.299^a^
- Median (IQR)	67 (60, 68)	60 (56, 66)	
Height (cm)			
- Mean ± SD	161.27 ± 9.45	159.80 ± 7.06	0.634^a^
- Median (IQR)	160 (150, 170)	160 (155, 165)	
BMI (kg/m^2^)			0.488^a^
- Mean ± SD	24.92 ± 2.95	24.18 ± 2.82	
- Median (IQR)	23.80 (22.20, 28.00)	23.40 (21.90, 26.20)	
Young and Burgess Classification			0.688^c^
- LC1	7	10	
- LC2	1	1	
- LC3	2	0	
- APC1	0	0	
- APC2	2	1	
- APC3	2	3	
- VS	1	0	
Surgery (Yes / No)	8/7	4/11	0.264^c^

*APC* Anteroposterior compression, *BMI* Body mass index, *IQR* Interquartile rage, *LC* Lateral compression, *SD* Standard deviation

^a^independent t-test

^b^Mann Whitney U Test

^c^Fisher’s Exact Test

The mean and median Hct at the ER, at 24 h, and the Hct change were comparable among groups. (Table 2) The amount of packed red blood cell transfusion and time to apply binder were statistically significant lower in the early pelvic sling group, although hospital stays and time to receive X-ray film were not. (Table 3) There were no problems associated to soft tissue and skin necrosis in either group of patients.

**Table 2:** Hematocrit

Hematocrit at ER	Early pelvic sling	Conventional pelvic sling	Mean diff.	95%CI	*p*-value
- Mean ± SD	33.48 ± 4.36	34.13 ± 6.57	–		0.751^a^
- Median (IQR)	33.10 (30.20, 35.90)	32.10 (28.80, 41.20)	–		
Hematocrit at 24 h					
- Mean ± SD	31.19 ± 2.98	32.00 ± 4.72			0.577^a^
- Median (IQR)	31.00 (28.70, 32.10)	31.10 (28.70, 36.50)			
Hematocrit Change					
- Mean ± SD	2.1 (0.6,4.2)	0.70 (1.3,5.2)	−1.50	−3.20-0.70	0.191

*SD* Standard deviation, *Mean diff.* Mean difference

^a^independent t-test

^b^Mann Whitney U Test

^c^Fisher’s Exact Test

**Table 3:** Packed red blood cell transfusion, Hospital stays, Time to receive X-ray film, Time to Apply binder

Outcomes	Early pelvic binder case	Conventional pelvic binder case	Mean diff.	95% CI	*p*-value
	Mean (SD)	Mean (SD)			
	*n* = 15	*n* = 15			
Packed red blood cell transfusion	0.80 ± 1.42	2.4 ± 2.32	–	–	0.008^a^
Hospital stays (day)	12 (8,15)	11 (8, 16)	− 1.00	−7.00 - 10.00	0.803^a^
Time to receive X-ray film (min)	33.33 ± 9.07	32.73 ± 12.93	0.60	−7.75 - 8.95	0.884^b^
Time to Apply binder (min)	16.40 ± 5.45	40.40 ± 13.64	−24.00	− 31.77 - -16.24	< 0.001^b^

*SD* Standard deviation, *Mean diff.* Mean difference

^a^Mann Whitney U Test

^b^independent t-test

## Discussion

Pelvic ring injuries have a high mortality and morbidity rate [11]. Shearing of pelvic vessels, as well as bleeding from fractured bone ends, can result in life-threatening retroperitoneal hemorrhage, adding to morbidity [12]. In cases of suspected pelvic fracture, one time pelvic compression test may be performed to identify laxity and instability, but this should be done with caution because a formed blood clot may dislodge, resulting in further hemorrhage [13]. Shlamovitz et al. discovered that the sensitivity of this test was only 8%, implying that it may be unreliable for detecting instability [14]. As a result, in the early stages of major trauma care, the presence of pelvic disruption should be suspected after considering the mechanism of injury rather than confirmed by physical examination [15].

Because of its efficiency in lowering pelvic volume for the tamponade effect [16], the use of a PCCD has become a standard part of Advanced Trauma Life Support protocol. Bakhshayesh et al. supported the use of the PCCD in the initial care of patients with suspected pelvic bleeding in their systematic review [17]. Despite the fact that Pap et al.’s recent study revealed that the PCCD is not clearly connected to improved clinical outcomes and may cause iatrogenic harm, the clinical advantages appear to exceed the hazards [17, 18].

Regarding the time to apply the PCCD, Vaidya et al. found that most of the PCCD were performed after imaging [19]. The sooner the bleeding is stopped, the better the chances of lowering the mortality rate. Our study discovered that the time to apply a PCCD in the early pelvic sling group compared to the conventional stepwise group was 16.40 and 40.40 min, respectively. Fu et al. demonstrated that employing a PCCD in patients with a pelvic ring injury who were transferred to another institution resulted in considerably lower transfusion requirements, regardless of whether they were hemodynamically stable or unstable prior to transfer [4]. In their retrospective cohort study, Hsu et al. demonstrated that patients receiving pelvic sling before definitive imaging had a significantly lower transfusion requirement [7]. Our findings were comparable in that the need for packed red blood cell transfusions was statistically lower in the early pelvic sling group (0.80 unit) than in the conventional group (2.40 units). However, the Hct change does not differ significantly between groups. According to Ryan et al., the admission Hct linked with hemorrhage in trauma patients requiring emergency surgery, but the Hct change did not [20]. This could be because the Hct change contains various confounders that are difficult to assess, such as IV hydration, blood transfusion, and continuous hemorrhage.

According to Ghaemmaghami et al., early application of PCCD may have limited utility in centers with quick access to angioembolization [6]. They did not, however, specify the number of each type of fracture. Our study attempted to address this issue by categorizing the types of pelvic fractures. Moreover, they also excluded patients with LC1 fractures and those with isolated ileal wing fractures. Although stable pelvic fractures are frequently regarded as minor injuries and are typically treated conservatively, 7–13.9% of patients with stable pelvic fractures require embolization for hemostasis [21, 22]. Takeda et al. reported a patient with stable pelvic ring fractures, hemorrhagic shock, and acute traumatic coagulopathy. The SAM Sling® was used to stop the hemorrhaging, and she was eventually rescued [23]. Early application of PCCD, in our opinion, would benefit the population with these types of fractures in terms of transfusion requirements because the associated hemorrhage from the fracture surface could be reduced with the compression force of the PCCD.

The PCCD can be used in both lateral compression (LC) and anterior posterior compression (APC) pelvic fractures [24], but there may be some debate in the vertical shear (VS) type. Despite the fact that we only had one patient with VS type in the early pelvic sling group, there was no consequence from the PCCD in this patient. Our findings matched those of Hsu et al., who conducted a retrospective investigation on 204 pelvic ring injuries [7]. The PCCD was used in various types of fractures with no complications from over-reduction. However, key drawbacks of the PCCD include the fact that they do not control VS fractures and do not stop arterial bleeding; thus, access to administer embolization is critical.

Although the use of the PCCD has been deemed safe due to its noninvasive nature, clinicians should be aware of the possibility of adverse consequences. Hsu et al. reported three patients who experienced complications because of wearing the pelvic binder for an extended period of time [7]. To reduce the risk of developing pressure sores, skin necrosis and nerve palsy, Knopps et al. suggested removing the pelvic sling once hemodynamic resuscitation was established [25]. The 24-h protocol for the early pelvic sling group in our study not only reduced packed red blood cell transfusion requirements statistically significantly compared to the conventional approach, but also revealed no complications due to soft tissue and skin necrosis. Suzuki et al. [26] and Toth et al. [5] also described major adverse effects from PCCD use, such as bladder rupture or external iliac vein compression. Because all complications were discovered after the PCCD was applied, it was difficult to distinguish between those caused by the original injury and those caused by the PCCD. Nevertheless, doctors should be aware of all of these complications, especially when treating patients with suspected acetabular fractures.

Our paper’s strength is its use of a force-controlled circumferential pelvic sling, which has been scientifically demonstrated to decrease and stabilize pelvic fractures safely and successfully [27]. It has a fastener with an auto-stop buckle that limits circumferential compression when tensional force exceeds 150 N. There are a few potential limitations to our study. It was a single-institution event that may have reflected local patient characteristics. As with most retrospective assessments, unmeasured or unknown confounding variables may be to blame for the effects observed and the conclusions reached. The sample size was small, with only 15 patients in each group. This may result in a failure to capture the full statistical significance of these factors. Finally, despite the fact that patients in both groups had similar demographic data, case allocation heterogeneity between the two arms was possible due to the recruitment method.

## Conclusion

Early application of the PCCD for 24 h before clinical and radiological confirmation has effectively lowered the rate of packed red blood cell transfusion in any pelvic fracture case without device-related complications. However, the Hct change does not differ significantly between groups.

## Data Availability

The datasets generated during and/or analyzed during the current study are available from the corresponding author on reasonable request.

## Abbreviation

GCS: Glasgow coma score
